# Comparison of outcomes in emergency department patients with suspected cardiac chest pain: two-centre prospective observational study in Southern China

**DOI:** 10.1186/s12872-018-0814-4

**Published:** 2018-05-16

**Authors:** Huilin Jiang, Yunmei Li, Junrong Mo, Xiaohui Chen, Min Li, Peiyi Lin, Kevin K. C. Hung, Timothy H. Rainer, Colin A. Graham

**Affiliations:** 1 Emergency Department, The Second Affiliated Hospital of Guangzhou Medical University, Guangzhou, China; 2 Accident and Emergency Medicine Academic Unit, Chinese University of Hong Kong, Prince of Wales Hospital, Main Clinical Block and Trauma Centre, Shatin, N.T, Hong Kong China

**Keywords:** Acute coronary syndrome, Risk stratification, Emergency department, Chest pain, Hong Kong, Guangzhou

## Abstract

**Background:**

Hong Kong (HK) and Guangzhou (GZ) are cities in China with different healthcare systems. This study aimed to compare 30-day and 6-month mortality and characteristics of patients with suspected cardiac chest pain admitted to two emergency departments (ED) in HK and GZ.

**Methods:**

A prospective observational study enrolled patients with suspected cardiac chest pain presenting to EDs in the Prince of Wales Hospital (PWH), HK and the Second Affiliated Hospital of Guangzhou Medical University (AHGZMU),GZ. The primary outcome was 30-day and 6-month mortality.

**Results:**

In total, 996 patients were recruited, 407 cases from GZ and 589 cases from HK.The 30-day and 6-month mortality of chest patients were 3.7% and 4.7% in GZand 0.3% and 1.9% in HK, respectively. Serum creatinine level (Cr) was an independent factor for 30-day mortality whilst Cr and systolic blood pressure (SBP) were independent factors for 6-month mortality. In Cox regression analysis, unadjusted and adjusted hazard ratios for 30-day and 6-month mortality in GZ were significantly increased.

**Conclusion:**

The 30-day and 6-month mortality of patients with **s**uspected cardiac chest pain in Guangzhou were higher than in Hong Kong due to due to different baseline clinical characteristics of patients and different distributions of diagnoses, which were associated with different healthcare systems. Serum creatinine and SBP were independent factors for 30-day and 6-month mortality.

**Electronic supplementary material:**

The online version of this article (10.1186/s12872-018-0814-4) contains supplementary material, which is available to authorized users.

## Backgroud

Chest pain is a common chief complaint of patients presenting to emergency departments (ED) globally, and it places a huge burden on ED services [1–3]. The challenge for clinicians is the dual danger of discharging patients at potential high risk and the clinical pressure of EDs crowded with low risk patients. In order to improve the quality of care and survival rates of cardiac patients, there is a worldwide impetus to develop and improve systems for emergency cardiac care [4]. Current guidelines emphasize rapid determination of the likelihood that the patient’s clinical presentation represents high risk chest pain, such as acute coronaryartery disease, and rapid assessment of the immediate risk for a major adverse cardiac event (MACE) [5]. Hong Kong (HK) and Guangzhou (GZ) are cities in southern China with almost the same ethnicity but different healthcare systems [6]. The healthcare system of HK has adopted health care financing and organizational health systems that are commonly seen in centrally planned economies. In contrast, mainland China has integrated many features of health care systems associated with market economies, whereas both have advanced systems of chest pain care in EDs [7–9]. So the healthcare systems of the two cities are quite different under the “one country, two systems” policy [10]. No study has compared clinical outcomes of susceptive cardiac chest pain patients with similar ethnicity under different healthcare systems, and the influence factor of its outcome. Our previous study has compared the clinical ED management and one-year mortality of ST-segment elevation myocardial infarction (STEMI) patients between the two hospitals in HK and GZ [11]. After that, the comparable registries of chest pain database have been developed in Guangzhou and Hong Kong. The objective of the study was to compare 30-day and 6-month outcomes of patients with suspected cardiac chest pain presenting to two EDs in HK and GZ, and contributory factors of outcomes.

## Methods

### Study design and setting

A prospective cohort study conducted from May 2012 to August 2013 in two teaching hospitals.

The Second Affiliated Hospital of Guangzhou Medical University (AHGZMU)is an academic hospital with 1500 beds affiliated with the Guangzhou Medical University and serves a population of approximately 1.56 million people in the Hai Zhu district, Guangzhou. The ED receives more than 150,000 new patients per annum and serves a local population of approximately 1,680,000 people. The Prince of Wales Hospital (PWH) is located in the New Territories in Hong Kong. It is a university hospital with 1400 beds. It sees approximately 150,000 new ED patients per annum and serves a local population of approximately 800,000 people.

### Inclusion and exclusion criteria

Consecutive ED patient ≥18 years old, with a chief complaint of chest pain or discomfort were recruited. Patients were excluded if they clearly had a non-cardiac cause of chest pain. Confirmed STEMI patients at ED presentation were also excluded as they did not have undifferentiated chest pain. Patients were excluded if they were unable or unwilling to provide informed consent or unable to be contacted after discharge.

### Measurements and data collection

Baseline patient characteristics, the characteristics of the chest pain (such as location, feature and radiation), vital signs, medical history, family history of coronary artery disease (CAD), ECG results and contact information to facilitate subsequent follow-up were collected and recorded in a computerized database. The HEART score was recorded and calculated by research staff. The details of the HEART score are shown in Additional file 1. Guidelines recommend that patients with possible ACS but with a normal initial ECG and cardiac markers should be observed in a chest pain unit, where continuous cardiac monitoring and repeated measurement of cardiac markers is available [12]. Point-of care troponin testswere used in GZ, whilst high sensitivity troponin tests were used in HK. Therefore, the results of troponin were divided into four groups (≤1× normal range, 1×-2× normal range, 2×-3× normal range and ≥ 3× normal range) for analysis. ST-depression on initialECG means ST depression ≥1.0mv in any lead of the initial ECG. The onset-ED time and ED-ECG time were recorded. Follow-up data were obtained from the Clinical Management System (CMS) in HK and Health Insurance Information Management System (HIIMS) in GZ.

### Follow up

Subsequent visits to ED, hospital readmission for evaluation of chest pain and all cardiac procedures performed were retrieved from CMS in HK and HIIMS in GZ, and verified via telephone at 30-days and 6-months follow-up after initial presentation. Furthermore, death, myocardial infarction, readmission for ACS, and all cardiac testing and coronary revascularization procedures were also obtained via CMS and HIIMS.

### Sample size calculation

According to our previous study [7], the 30-day MACE rates in Chinese patients presenting to ED with cardiac chest pain could have been as high as the upper limit of the 95% confidence interval in the low-risk group i.e. 13%, and as low as the lower limit of the 95% confidence interval in the high-risk group i.e. 23%. To achieve adequate power to address the objectives by using 2-tailed alpha of 0.05 and a power of 80%, the minimum sample size required per group is 220. We aimed to recruit an extra 30% in case of unforeseen circumstances and thus at least 230 (230 × 1.3 = 299) patients were required per group. Therefore, the minimum sample size in this study was 598 (299 × 2 recruiting sites = 598).

### Outcomes

The primary outcomefor each patient wasall cause mortality within 30 days and 6 monthsafter initial ED presentation. The secondary outcomes were the contributory factors of primary outcomes.

### Statistical analyses

Continuous variables were presented as median and interquartile range or mean ± standard deviation as appropriate. Categorical variables were expressed as frequencies. Statistical analysis was performed using SPSS v17.0 (SPSS Inc., IL, USA) and Medcalc v9.5 (MedCalc Software, Mariakerke, Belgium). Summary statistics were used to describe patient characteristics from the Hong Kong and Guangzhou groups. Chi-square analysis was used for categorical variables, whilst independent t-tests or Mann-Whitney U-tests were used for comparing data from continuous variables. An initial univariate analysis was performed on all variables with 30-day and 6-month mortalityas the dependent variables and presented as unadjusted odds ratios (OR) and 95% confidence intervals (95% CI). Variables with *p* ≤ 0.1 were entered into a multivariate ordinal logistic regression. Cumulative survival curves in relation to different hospitals were determined according to the Kaplan-Meier method with the use of log-rank tests for statistical assessment. Cox regression analysis was used to calculate unadjusted and adjusted hazard ratios (HRs) and 95% CIs for mortality rates after 30 days and 6 months in relation to different hospitals. A *P* value of < 0.05 was considered statistically significant and all probabilities were two tailed.

## Results

### Recruitment of patients from the two centers

**Fig. 1:**
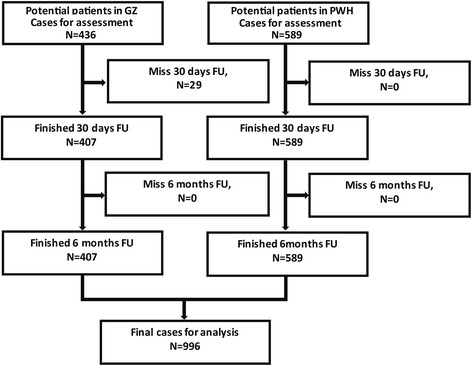
Flow chart of eligible patients in the two hospitals

Patients were recruited from 17 March 2012 to 14 August 2013. The flow chart of the patient recruitment was shown in Fig. 1. There were 1025 eligible patients, with 589 cases in HK and 436 cases in GZ. Twenty-nine patients were excluded due to lack of follow-up data, leaving 996 patients (407 cases from GZ and 589 cases from HK) in the study. The mean age of the 996 patients was 65.1 ± 14.5 years and 55.3% were males.

### Baseline characteristics and final diagnoses of chest pain patients

Table 1 shows the characteristics of chest pain patients from GZ and HK. Compared to patients in HK, patients in GZ were older and had lower proportions of past history of diabetes, hyperlipidaemia and chronic heart failure, lower proportion of NHYA class I and lower blood pressure. Troponin is a key biomarker to detect myocardial injury in the two hospitals. Troponin was used as a myocardial injury biomarker in 98% patients in HK and 70% patients in GZ. Creatine kinase (CK) and creatine kinase isoenzyme-MB (CK-MB) were the predominant biomarkers used in GZ, in which 98% patients had a CK level determined. However, these tests were rarely used in HK. Ischaemic cardiac chest pain was diagnosed in 120 (29.4%) patients in GZ (9.8% with NSTEMI, 16.0% with unstable angina and 3.7% with stable angina). Non-ischaemic cardiac aetiologies and non-cardiac aetiologies were found in 136 (33.4%) and 151 (37.1%) patients respectively. In HK, 163 (27.7%) patients had ischaemic cardiac chest pain, including 5.4% with NSTEMI, 12.1% with unstable angina and 10.2% with stable angina. 84 (14.3%) patients were diagnosed as having non-ischaemic cardiac aetiologies and 342 (58.1%) were diagnosed with non-cardiac aetiologies. There were significant differences in the number of cases diagnosed with non-ischaemic cardiac aetiologies and non-cardiac chest pain between GZ and HK (*P* < 0.0001), whilst there was no significant difference in the number of cases diagnosed with ischaemic cardiac chest pain between the two hospitals.There were no significant differences in the number of readmission of 30-day and 6-month.

**Table 1:** Comparison of patient characteristics between the two hospitals (*N* = 996)

	AHGZMU (*n* = 407)	PWH (*n* = 589)	*P*
Age, years, mean (SD)	66.6 (14.4)	63.7 (14.6)	0.002*
Males, *n* (%)	220 (54.1)	325 (55.2)	0.726
Past histories			
Smoker, *n* (%)	69 (17.0)	78 (13.2)	0.105
Hypertension, *n* (%)	245 (60.2)	362 (60.9)	0.688
Diabetes, *n* (%)	79 (19.4)	153 (26.0)	0.016*
Hyperlipidemia, *n* (%)	92 (22.6)	266 (45.2)	< 0.0001*
Chronic heart failure, *n* (%)	26 (6.4)	62 (10.5)	0.024*
Family heart disease, *n* (%)	72 (17.7)	125 (21.2)	0.169
Stroke, *n* (%)	26 (6.4)	72 (12.2)	0.002*
Peripheral arterial disease, *n* (%)	6 (1.5)	20 (3.4)	0.062
NHYA classification			
NHYA class I, *n* (%)	234 (57.5)	401 (68.1)	0.001*
NHYA class II, *n* (%)	118 (29.0)	75 (12.7)	< 0.0001
NHYA class III, *n* (%)	51 (12.5)	108 (18.3)	0.014*
NHYA class IV, *n* (%)	4 (1.0)	5 (0.8)	0.826
First ED characteristics			
Heart rate, bpm, mean (SD)	88.4 (27.8)	82.0 (19.1)	< 0.0001*
Systolic blood pressure, mmHg, mean (SD)	141.3 (30.1)	148.7 (25.9)	0.001*
Diastolic blood pressure, mmHg, mean (SD)	78.2 (17.0)	80.1 (15.4)	0.018
First lab results in ED			
Creatinine, μmoL/L, median (IQR)	88.0 (71.0, 109.0)	81.0 (66.0, 97.0)	< 0.0001*
Troponin			
≤ 1×	224 (78.9)	420 (72.4)	< 0.0001*
1×-2×	21 (7.4)	85 (14.7)	< 0.0001*
2×-3×	8 (2.8)	22 (3.8)	0.108
≥ 3×	31 (10.9)	53 (9.1)	0.441
Number of CK done, *n* (%)	399 (98.0)	339 (57.6)	< 0.0001*
Number of CK-MB done, *n* (%)	399 (98.0)	0 (0)	< 0.0001*
ST segment depression, *n* (%)	80 (8.0)	19 (1.9)	< 0.0001*
Onset- ED time,hour, median (IQR)	4.03 (1.5–13.21)	6.52 (2.01–23.27)	< 0.0001*
ED-ECG time, hour, median (IQR)	0.17 (0.03–0.47)	0.18 (0.12–0.28)	0.234
HEART Score, median (IQR)	4 (3,6)	4 (2,5)	< 0.0001*
Number of admission	231 (56.8)	334 (56.7)	0.987
Final diagnoses			
Ischemia coronary chest pain	120 (29.5)	163 (27.7)	0.534
NSTEMI, *n* (%)	40 (9.8)	32 (5.4)	
Unstable angina, *n* (%)	65(16.0)	71(12.1)	
Stable angina, *n* (%)	15 (3.7)	60 (10.2)	
Non-ischaemia cardiac chest pain, *n* (%)	136 (33.4)	84 (14.3)	< 0.0001*
Non-cardiac chest pain, *n* (%)	151 (37.1)	342 (58.1)	< 0.0001*

*statistically significant

### Comparison of clinical outcomes of chest pain patients in the two centres

Table 2 shows the 30-day and 6-month mortality of chest painpatients. The 30-day mortality rates for ischaemia, non-ischaemic cardiac and non-cardiac causes in GZwere 1.5%, 1.0% and 1.7% respectively. No one died of ischaemia and non-ischaemia cardiac aetiologies in HK up to 30-days follow-up. The 30-day mortality rates for non-cardiac causes was 0.7% in HK. The 6-month mortality rates of ischaemic chest painin GZ were statistically higher than those in HK.

**Table 2:** Comparison of 30-day and 6-month mortality in patients with different diagnoses between the two hospitals (*N* = 996)

	30-day mortality in GZ (*n* = 407)	30-day mortality in HK (*n* = 589)	*P*	6-month mortality in GZ (*n* = 407)	6-month mortality in HK (*n* = 589)	*P*
Final diagnoses						
Ischemia coronary chest pain	6 (1.5)	0 (0)	0.005*	7 (1.7)	2 (0.3)	0.036*
NSTEMI, *n* (%)	4 (1.0)	0 (0)		5 (1.2)	1 (0.2)	
Unstable angina, n (%)	2 (0.5)	0 (0)		2 (0.5)	0 (0)	
Stable angina, *n* (%)	0 (0)	0 (0)		0 (0)	1 (0.2)	
Non-ischaemia cardiac causes, *n* (%)	4 (1.0)	0 (0)	0.28	5 (1.2)	3 (0.5)	0.283
Non-cardiac causes, *n* (%)	5 (1.2)	2 (0.3)	0.129	7 (1.7)	6 (1.0)	0.339
Total	15 (3.7)	2 (0.3)	< 0.0001*	19 (4.7)	11 (1.9)	0.011

The death reasons of non-cardiac chest pain were: pulmonary embolism = 1 in GZ, pneumonia = 1in GZ and 3 in HK, ischaemic stroke = 1 in GZ, cancer = 1 in GZ and 3 in HK, other reasons = 3 in GZ

The death reasons of non-ischaemic cardiac chest pain were: heart failure = 2in GZ and 2 in HK, Valvular heart disease = 1 in HK, Aortic dissection = 3 in GZ

*statistically significant

The 6-month mortality of ischemia, non-ischaemic cardiac and non-cardiac causes in GZ were 1.7%, 1.2% and 1.7% respectively, whilst in HK these were 0.3%, 0.5% and 1.0% respectively. NSTEMI patients demonstrated a higher mortality than other ischaemic chest pain patients at 30-day and 6-month follow-up. Compared with cardiac aetiologies, higher mortality was observed for non-cardiac causes. Most diagnoses of non-cardiac chest pain were pulmonary embolism, pneumonia, ischemia stroke and cancer (see Table 2 for details).

### Univariate and multivariate logistic regression for 30-day and 6-month mortality

Table 3 shows patients were more likely to die after 30 days if they were older, had ST-segment deviation, a history of worse NHYA class and lower SBP and DBP, higher serum creatinine and troponin level at ED admission, and a higher HEART score. After multivariate logistic regression, serum creatinine level was the only independent predictor of 30-day mortality.

**Table 3:** Univariate and multivariate logistic regression for 30-day mortality (*N* = 996)

	Unadjusted Odds Ratio (95% CI)	*P*	Adjusted Odds Ratio (95% CI)	*P*
Age, years	1.069 (1.023–1.117)	0.003*	1.031 (0.977-1.089)	0.262
Males (vs Females)	2.008 (0.702–5.744)	0.193		
Past histories				
Smoker (vs non smoker)	2.256 (0.644–7.905)	0.204		
Hypertension (vs non hypertension)	1.094 (0.413–2.899)	0.857		
Diabetes (vs non diabetes)	0.702 (0.2–2.464)	0.58		
Hyperlipidemia (vs non hyperlipidemia)				
CHF (vs non CHF)	2.254 (0.635–7.998)	0.209		
FHD (vs non FHD)	1.153 (0.328–4.053)	0.824		
Stroke (vs non stroke)	0.568 (0.075–4.332)	0.586		
PAD (vs non PAD)				
First ED characteristics				
Heart rate	1.003 (0.982–1.024)	0.804		
SBP	0.980 (0.962–0.990)	0.044*	0.990 (0.963-1.089)	0.486
DBP	0.973 (0.942–1.005)	0.1	1.02 (0.97–1.072)	0.435
NHYA class III, IV (vs NHYA class I,II)	3.557 (1.334–9.481)	0.011*	1.554 (0.405-5.972)	0.521
Troponin Elevation ≥2× (vs < 2×)	10.385 (1.228–87.874)	0.032*	2.471 (0.613-9.955)	0.203
Creatinine elevation (vs normal)	11.643 (3.947–34.347)	< 0.0001*	9.189 (2.319-36.407)	0.002*
ST segment depression (vs normal)	0.175 (0.061–0.5)	0.001*	1.026 (0.973-1.083)	0.34
HEART Score	1.622 (1.228–2.143)	0.001*	0.706 (0.404-1.231)	0.22
Onset- ED time	0.966 (0.919–1.015)	0.172		
ED-ECG time	1 (1–1)	0.949		

*CHF* Chronic heart failure, *FHD* Family history of heart disease *PAD* Peripheral arterial disease, *SBP* Systolic blood pressure, *DBP* Diastolic blood pressure

Adjusted for age, SBP, DBP,ST segment depression, NHYA classification, troponin, HEART score and serum creatinine

*statistically significant

Table 4 shows patients were more likely to die by 6 months if they were older, had ST-segment depression, a history of chronic heart failure, hyperlipidemia and worse NHYA class, lower SBP, higher serum creatinine and troponin level at ED admission, and higher HEART score. After multivariate logistic regression, serum creatinine level and SBP were the only independent predictors of 6-month mortality.

**Table 4:** Univariate and multivariate logistic regression for 6-month mortality (*N* = 996)

	Unadjusted Odds Ratio (95% CI)	*P*	Adjusted Odds Ratio (95% CI)	*P*
Age, years	1.074 (1.038–1.112)	< 0.0001*	1.033 (0.98-1.088)	0.233
Males (vs Females)	0.508 (0.230–1.121)	0.093	0.592 (0.17–2.053)	0.592
Past histories				
Smoker (vs non smoker)	0.755 (0.332–1.715)	0.502		
Hypertension (vs non hypertension)	1.291 (0.598–2.789)	0.515		
Diabetes (vs non diabetes)	1.002 (0.425–2.367)	0.996		
Hyperlipidemia (vs non hyperlipidemia)	0.191 (0.058–0.635)	0.007*	0.00 (0.00-0.00)	0.996
CHF (vs non CHF)	4.812 (2.132–10.861)	< 0.0001*	3.454 (0.705-16.93)	0.126
FHD, (vs non FHD)	0.442 (0.133–1.473)	0.184		
Stroke (vs non stroke)	1.019 (0.303–1.473)	0.976		
PAD (vs non PAD)	1.298 (.170–9.909)	0.801		
First ED characteristics				
Heart rate	1.007 (0.993–1.022)	0.318		
SBP	0.979 (0.965–0.993)	0.004*	0.974 (0.95-0.999)	0.045*
DBP	0.977 (0.954-1.001)	0.061	1.045 (0.998–1.094)	0.114
NHYA class III, IV (vs NHYA class I,II)	5.314 (2.545–11.095)	< 0.0001*	2.519 (0.685-9.266)	0.165
Troponin Elevation ≥ 2× (vs < 2×)	8.04 (1.693–38.185)	0.009*	2.983 (0.494-18.01)	0.233
Creatinine elevation (vs normal)	11.137 (4.23–29.326)	< 0.0001*	7.48 (2.042-27.369)	0.002*
ST segment depression (vs normal)	4.190 (1.968–8.917)	< 0.0001*	1.006 (0.954-1.061)	0.815
HEART Score	1.603 (1.310–2.027)	< 0.0001*	0.81(0.487-1.349)	0.418
Onset- ED time	0.986 (0.964–1.008)	0.216		
ED-ECG time	1.00 (1.000–1.000)	0.922		

*CHF* Chronic heart failure, *FHD* Family history of heart disease, *PAD* Peripheral arterial disease, *SBP* Systolic blood pressure, *DBP* Diastolic blood pressure

Adjusted for age, gender, hyperlipidemia, CHF, SBP, DBP, ST segment depression, NHYA classification, troponin, HEART score and serum creatinine

*statistically significant

### Survival analysis in two hospitals

**Fig. 2:**
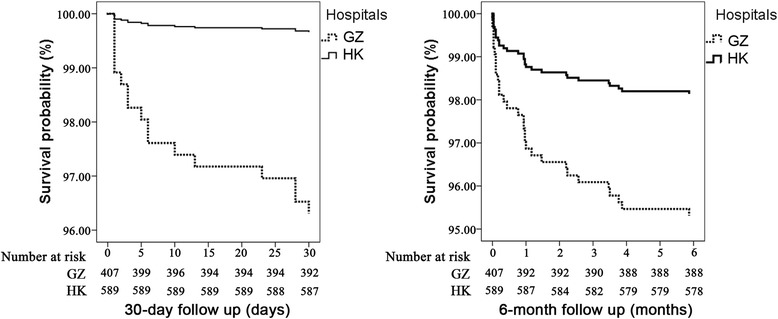
Kaplan-Meier event-free survival curves in GZ and HK. Kaplan-Meier analysis demonstrated a significant increased probability of mortality during 30-day (Log Rank *P <* 0.0001) and 6-month follow-up in GZ (Log Rank *P* = 0.010)

Figure 2 showed the corresponding Kaplan-Meier event-free survival curves for different hospitals. Kaplan-Meier analysis demonstrated a significantly increased probability of death ofchest pain patients in GZ after 30 days and 6 months (*P* < 0.0001 for 30-day mortality using the log-rank test, and *P* = 0.010 for 6-month mortality using the log-rank test).

In Cox regression analysis, the unadjusted HR for 30-day (HR = 11.05, 95% CI 2.53 to 48.32, *P* = 0.001) and 6-month (HR = 2.55, 95% CI 1.22 to 5.37, *P* = 0.013) mortality in GZ was significantly higher (Table 5). After adjusting for age, SBP, ST segment depression, NHYA classification, troponin, HEART score and serum creatinine, the adjusted HR for 30-day (HR = 8.98, 95% CI 1.78 to 45.2, *P* = 0.008) mortality in GZ was still significantly higher.

**Table 5:** Unadjusted and adjusted hazard ratios of all-cause mortality at 30-days and 6-months in the two hospitals

	HR(95% CI)	*P*	Adjusted HR (95% CI)	*P*
30-day all-cause mortality				
GZ	11.05 (2.53–48.32)	0.001*	8.98 (1.78-45.2)	0.008*
HK	Reference		Reference	
6-month all-cause mortality				
GZ	2.55 (1.22–5.37)	0.013*	1.77 (0.73-4.26)	0.205
HK	Reference		Reference	

30-day mortality was adjusted for age, SBP, DBP, ST segment depression, NHYA classification, troponin, HEART score and serum creatinine

6-month mortality was adjusted for age, gender, hyperlipidemia, CHF, SBP, DBP, ST segment depression, NHYA classification, troponin, HEART score and serum creatinine

*statistically significant

After adjusting for age, hyperlipidemia, CHF, SBP, ST segment depression, NHYA classification, troponin, HEART score and serum creatinine, the adjusted HR for 6-month (HR = 1.77, 95% CI 0.73 to 4.26, *P* = 0.205) mortality in GZ was not significantly higher than that in HK.

## Discussion

This is the first prospective cohort study to compare 30-day and 6-month mortality of suspected cardiac chest pain patients admitted to EDs between two teaching hospitals located in two different cities in southern China with similar ethnicity but differenthealthcare systems [11]. The chest pain patients in GZ had a higher 30-day and 6-month mortalitythan in HK. The findings suggest that the different outcomes were associated with the varying clinical characteristics of patients and different distributions of diagnoses under two differenthealthcare systems. GZ has integrated many features of healthcare system associated with market economies, while its overall economy is largely centrally planned. In contrast,Hong Kong adopt thehealthcare system are come from England and organizational health systems that are commonly seen in centrally planned economies, while its economy functions as a highly capitalisticenterprise [10, 13].

### Different distributions of chest pain patients between two hospitals

Over 50% of patients were diagnosed as non-cardiac chest pain in HK, a much higher rate than in GZ (37.1%). This may reflect the different healthcare systems. In Guangzhou, chest pain patients at low risk often present to clinics instead of EDs during office hours. The ED is the only choice for patients with chest pain in HK due to lower medical costs. So, the patients with urgent and severe chest pain/symptoms would likely to present to EDs in GZ. Compared to other studies, the proportion of non-cardiac chest pain patientsin this study was low. The proportions in GZ and HK were 50% and 37.1% respectively compared to 67.2%–70% in other studies [14–16]. A lower proportion of non-cardiac chest pain patients may allow earlier discharge from the ED and place less stress on ED and hospital services.

There were no significant differences in 30-day and 6-month mortality for non-ischemic cardiac chest pain and non-cardiac chest pain between the two hospitals. However, there was significantly higher 30-day and 6-month mortality rates for ischemic cardiac chest pain patients in GZ. ACS is a high-risk disease with significant mortality [9, 17]. The current study excluded STEMI patients but NSTEMI and UA patients were recruited. The combined proportion of NSTEMI and UA in GZ was 25.8%, which was higher than thatin HK (17.5%). The proportions of NSTEMI and UA were also higher than those in Cullen’s study (8.7%) whilst non ACS cardiovascular-related conditions were 20.8% [14].

### Different clinical characteristics and outcomes of patients between two hospitals

Since the patients with severe chest pain/symptoms would likely to present to EDs in GZ while those patients with mild chest pains/symptoms would visit clinics, so the baseline clinical characteristicsof patients in GZ were worse than those in HK. The patients were older, had lower proportion of NHYA class I and higher creatinine level, higherproportion of ST-segment depression, lower proportion of negative TnT and higher HEART scoreat ED presentation. Those factors have also been shown to be associated with 30-day and 6-month mortality. The same parameters have been verified to predict short- and long-term risks of death from ACS [18–24]. Compared to HK, lower proportions of patients with risk factors were seen in GZ, whilst more patients with worse NHYA class were observed. This suggests that many chest pain patients in GZ did not have any knowledge of cardiac risk factors and were unaware of the presence of those risk factors, therefore the impact of those risk factors might be underestimated. Early detection and good control of risk factors for suspected cardiac chest pain patients may reduce mortality.

Furthermore, elevated serum creatinine level was an independent predictor for 30-day and 6-month mortality in our study.It has also been reported to be associated with worse outcomes for ACS and critically ill patients [18, 23]. Elevated serum creatinine can reflect vascular damage, renal impairment, endothelial dysfunction and impaired myocardial blood flow [24–27]. A multinational registry study by Tang et al. showed that SBP could predict the 6-month mortality of all subsets of ACS. As SBP increases by 10 mmHg, the hazard ratio would decrease by 0.95 [27].

Several studies have reported that the HEART score is not only related to the severity of chest pain in EDs but also predicts the occurrence of clinical endpoints [28–32]. Our study also demonstrated that the HEART score was positively associated with 30-day and 6-month mortality of suspected cardiac chest pain patients in the ED setting.

The dissimilarity of healthcare systems in the two cities is another reason for the different ED management. Limited capacity of hospital emergency care services, high out-of-pocket expenses with the need for up-front payment, prolonged discussions with the patient and families for both obtaining consent and pooling funds are likely to be major contributing factors for the long pre-hospital, in-hosptial delay and refused some expensive treatments [13, 32].

In ED chest pain pathways, guidelines recommend that an ECG should be recorded within 10 min and myocardial injury biomarkers should be measured as soon as possible in all suspected cardiac chest pain patients [4, 9]. The ED-ECG time and the proportion of patients having myocardial injury biomarkers measured in both hospitals met these guidelines.Myocardial injury biomarkers, such as troponin, CK and CK-MB are used to identify myocardial injury [33]. Many studies have shown that troponinis superior to CK and CK-MB as a biomarker for detection of myocardial injury [19, 20]. Therefore, troponin testing is recommended in most guidelines for its high sensitivity and specificity [17]. However in GZ, CK and CK-MB are still mainly used for the diagnosis of AMI and ACS due to their lower costs. High expenses with the need for up-front payment of troponin testing are likely to be a major contributing factor for the lower rate of use of troponin testing in GZ. The lower sensitivities and specificities of CK and CK-MB might contribute to the delayed diagnoses of AMI/ACS and later treatment of patients, possibly contributing to the higher early mortality in GZ. Except the mortaliy, readmissions after ACS are associated with higher long-term all-cause mortality [34].However, our study did not show the significant difference of 30-day and 6-month readmission rate in in HK and GZ. The reason was that the patients we enrolled were suspected cardiac chest pain patients instead of ACS.

### Strengths and limitations

The strengths of this study include the fact it is the first comparison of outcomes in different healthcare systems in a similarly populated region of southern China, which effectively eliminates many confounding factors in any comparison. The current study also has some limitations, including the sample size of this study which met our precalculated size but may still be considered small by international standards. Secondly, patient risk factors (i.e. the past medical histories) may have been underestimated due to variations in history taking between the two centres. Thirdly, as a prospective observational study, propensity score with matching would attempt to reduce the bias due to confounding variables. However, this method requires large sample size. Also, the procedure only controls for observed variables, so any hidden bias due to latent variables may remain after matching.Logistic regression and Cox regression can meet the need of this study.

## Conclusion

The 30-day and 6-month mortality of patients with suspected cardiac chest pain in Guangzhou were higher than in Hong Kong due to different baseline clinical characteristics of patients at ED presentation and different distributions of diagnoses which were associated with different healthcare systems. Serum creatinine and SBP were independent predictors of 30-day and 6-month mortality. Improved patient knowledge of risk factors along with early detection and good control of risk factors may lead to better outcomes for cardiac chest pain patients in southern China.

## Additional file

Additional file 1: Definition of HEART score [29]: There were five elements: history, ECG, age,risk factors and troponin in HEART score. Each variable was scored as 0, 1 or 2 points. Each patient will receive a score of 1-10. (DOCX 32 kb)

## Abbreviations

ACS: Acute coronary syndrome
AHGZMU: The Second Affiliated Hospital of Guangzhou Medical University
CABG: Coronary artery bypass grafting
CAD: Coronary artery disease
CK: Creatine Kinase
CK-MB: Creatine Kinase Isoenzyme-MB
CMS: Clinical Management System
cTnT: Cardiac troponin T
ED: Emergency department
GZ: Guangzhou
HK: HongKong
IQR: Interquartile range
MACE: Major adverse cardiac events
NSTEMI: Non ST-segment elevation myocardial infarction
PPCI: Primary percutaneous coronary intervention
PWH: Prince of Wales Hospital
STEMI: ST elevation myocardial infarction
US: United States
