# Supplementary material for: Comparison of outcomes in emergency department patients with suspected cardiac chest pain: two-centre prospective observational study in Southern China
Source: BMC Cardiovasc Disord. 2018 May 16;18:95. doi: 10.1186/s12872-018-0814-4 (PMC5956813; doi:10.1186/s12872-018-0814-4)
Supplement: Supplementary file 1 — Definition of HEART score [29]: There were five elements: history, ECG, age,risk factors and troponin in HEART score. Each variable was scored as 0, 1 or 2 points. Each patient will receive a score of 1-10. (DOCX 32 kb) [file 12872_2018_814_MOESM1_ESM.docx]

Additional file 1. Definition of HEART score [29]: There were five elements: history, ECG, age,risk factors and troponin in HEART score. Each variable was scoredas0,1 or 2 points. Each patient will receive a score of 1-10.

| Variables | | Descriptions | Score |
| --- | --- | --- | --- |
| History | Highly suspicious | History of typical ACS symptoms included    Characteristics: pressure, aching, burning    Location: substernal, left chest, left arm    Radiation: left arm, neck    Associated: diaphoresis, shortness of breath, vomiting | 2 |
|  | (>4 symptoms)  Moderately suspicious  (3–4 symptoms) |  | 1 |
|  | Slightly suspicious (0–2 symptoms) |  | 0 |
| ECG | Significant ST-depression | ST elevation≥1mm | 2 |
|  | Nonspecific repolarisation disturbance | T-wave inversion, ST depression or hyperacute T waves | 1 |
|  | Normal | Normal or nonspecific | 0 |
| Age | > 65 years |  | 2 |
|  | 45 – 65 years |  | 1 |
|  | < 45 years |  | 0 |
| Risk Factors | ≥ 3 risk factors or history of atherosclerotic disease | Risk factors :DM, current smoker, HT, hypercholesterolemia, family history of coronary artery disease, BMI≥30, history of significant atherosclerosis (coronary revascularization, myocardial infarction, stroke, peripheral arterial disease) | 2 |
|  | 1 or 2 risk factors |  | 1 |
|  | No known risk factors |  | 0 |
| Baseline Troponin | > 3x normal limit |  | 2 |
|  | 1 – 3x normal limit |  | 1 |
|  | < normal limit |  | 0 |

ACS: Acute coronary syndrome, DM: Diabetes mellitus, HT:Hypertension, BMI:Body mass index
